# Tat-dependent protein translocation and its evolution

**DOI:** 10.1093/femsre/fuag052

**Published:** 2026-09-22

**Authors:** Thomas Brüser, Carsten Sanders

**Affiliations:** Institute of Microbiology, Leibniz University Hannover, 30419 Hannover, Germany; Department of Biology, Millersville University, Millersville, PA 17551, USA; Biology and Chemistry Programs, School of Science and Mathematics, HACC Central Pennsylvania’s Community College, Harrisburg, PA 17110, USA; Department of Chemistry and Biochemistry, Saint Joseph’s University – Lancaster Campus, Lancaster, PA 17601, USA

**Keywords:** twin-arginine translocation, protein transport, membrane proteins, protein interactions, biological membranes, last universal common ancestor

## Abstract

The Tat system is a general translocation machinery responsible for the transport of folded proteins across biological membranes that are energized by a proton or sodium motive force. Protein substrates are characterized by usually amino-terminal signal peptides harboring a conserved twin-arginine sequence motif. Phylogenetic analyses suggest that a Tat system was already present in the last universal common ancestor. Today, Tat systems are found in bacteria, archaea, and bacteria-derived organelles. Minimally, Tat systems are composed of one TatC-family component and one TatA/B-family component, with multiple copies of these components forming the functional translocon, while more complex Tat systems exist that require additional homologs of TatA/B or TatC. Due to its ability to translocate folded proteins with bound cofactors, the Tat system is essential for a wide range of important physiological pathways, such as many anaerobic respirations or photosynthesis. With a focus on evolutionary aspects, and by including recent structural and mechanistic insights, we herein review our current knowledge on this mechanistically unique translocation pathway.

## Teaser

This review describes our current knowledge on Tat-dependent translocation of folded proteins and focuses on the evolution of this pathway.

“Nothing in Biology Makes Sense Except in the Light of Evolution.”

— Theodosius Dobzhansky (1973)

## Introduction

The twin-arginine translocation (Tat) pathway has the challenging function to transport folded proteins across biological membranes. This is the key functional difference to the Sec pathway, the general translocation route for proteins that fold after transport (Natale et al. 2008). Both systems date back to the last universal common ancestor (LUCA) and are found in all three domains of life (Cao and Saier 2003, Lewis and Hegde 2021, Yen et al. 2002). So far analyzed Tat systems function in membranes that are energized by ion gradients, such as the cytoplasmic membrane of bacteria and archaea, the thylakoid membrane of plant plastids, as well as the inner membrane (IM) of mitochondria in plants and several other eukaryotic lineages (Frain et al. 2019, New et al. 2018, Pohlschröder et al. 2004). Like the Sec system, the Tat system is a “general” translocation system in the sense that it is able to transport a wide range of proteins, even heterologous proteins that are recombinantly produced (Frain et al. 2019). There are only few requirements to change a folded protein to a Tat substrate: It requires on one hand a usually amino (N)-terminal Tat-specific signal peptide, which includes the eponymous “twin-arginine (RR) motif”, and on the other hand the absence of exposed larger hydrophobic regions, which is why the Tat system is suitable for the biotechnological production or genetic selection of proteins with correctly folded structures or improved solubility (Brüser 2007, Fisher et al. 2011). Natural Tat substrates are globularly folded proteins or protein domains ranging in size from <10 kDa, as in case of some monomeric high-potential iron sulfur proteins (HiPIPs), to over 140 kDa, as in case of the formate dehydrogenase α/β subunit complexes (Brüser et al. 1998, Stanley et al. 2002). Thus, Tat substrates have often dimensions larger than the membrane thickness, and the high tolerance of Tat transport with respect to size and shape of the cargo proteins is astonishing and fascinates researchers since the beginning of the 1990s (Hou and Brüser 2011). However, not only the structural characteristics of the Tat translocon have been puzzling, but also the energization of the translocation process. While Sec transport requires nucleotide hydrolysis and is further facilitated by a proton motive force (pmf) (Driessen and Wickner 1991, Shiozuka et al. 1990, Tsukazaki and Nureki 2011), Tat transport can be exclusively energized either by a pmf, such as in the model Tat systems of plant plastids or *Escherichia coli*, or by a sodium motive force (smf), as shown for halophilic archaea (Bageshwar and Musser 2007, Kwan et al. 2008, Theg et al. 2005). Tat transport is energetically expensive and these high energetic expenses are an inherent property of Tat systems at energized membranes. In plant systems, the number of 78 000 consumed protons per transported protein has been experimentally determined (Alder and Theg 2003). From this perspective, it is understandable that Tat systems are thought to be used for protein translocation only when folding needs to occur prior to translocation, for example, due to the requirement of cytoplasmic chaperones or cofactor insertion pathways (Berks 1996, Graubner et al. 2007, Turner et al. 2004). It could also be that easily achieved membrane energization may have contributed to a preferred use of Tat systems, as in cases of photosynthetic membranes in plant thylakoids or bacteriorhodopsin-containing membranes of haloarchaea (Peltier et al. 2002, Rose et al. 2002). Beside haloarchaea, which use the Tat system for most secreted proteins likely due to a required cytoplasmic folding at high salt concentrations (Rose et al. 2002), also streptomycetes and some genera of α-, β-, and γ-proteobacteria are predicted to transport up to 20% of their secretome via the Tat pathway (Dilks et al. 2003). It has not been clarified though, why in some of these organisms, such as *Caulobacter crescentus* or *Streptomyces coelicolor*, the majority of the proteins that need to be translocated by the Tat system are enzymes without redox cofactors (Dilks et al. 2003, Widdick et al. 2006). However, probably due to the large energetic costs of Tat transport, Tat systems were lost multiple times in evolution, especially when organisms no longer required a transport of folded redox proteins, as in case of many obligate fermentative organisms or obligate parasitic organisms (Dilks et al. 2003, Wu et al. 2000). In mitochondria, losses of Tat systems occurred multiple times as well (Petrů et al. 2018). Notably, in these mitochondria, nature evolved the specific ATPase Bcs1 of the AAA + family that substituted Tat systems for transport of the mitochondrial Rieske protein (Rosales-Hernandez et al. 2025, Wagener and Neupert 2012), which was most probably the only remaining Tat substrate, and the evolution of a specific ATPase surely was energetically cheaper than a Tat system with only one substrate.

In this review, we provide a comprehensive survey of the current knowledge on Tat system components and the associated translocation mechanism, before we focus in more depth on the evolutionary aspects. Our extensive search into the origin of Tat systems and their evolutionary diversity led to surprising points and considerations that we expect to shape future research.

### The Tat system components

The Tat system minimally consists of multiple copies of a TatC and a TatA/B family protein (Jongbloed et al. 2004). TatA/B family proteins have an N-terminal transmembrane (TM) helix with an N-out-topology that is followed by a short hinge that connects this helix to an amphipathic helix (APH) on the cis side of the membrane (Hu et al. 2010, Koch et al. 2012, Zhang et al. 2014) (Fig. 1A). The carboxy- (C-)terminal region of TatA/B family proteins is neither conserved in sequence nor required for translocation, whereas the membrane-anchor, the APH and several neighboring residues are essential (Lee et al. 2002, Warren et al. 2009). TatC is a polytopic membrane protein with, in most cases, six TM helices (TM1–TM6) and both termini on the cis side (Behrendt et al. 2004, Ramasamy et al. 2013, Rollauer et al. 2012).

**Figure 1:**
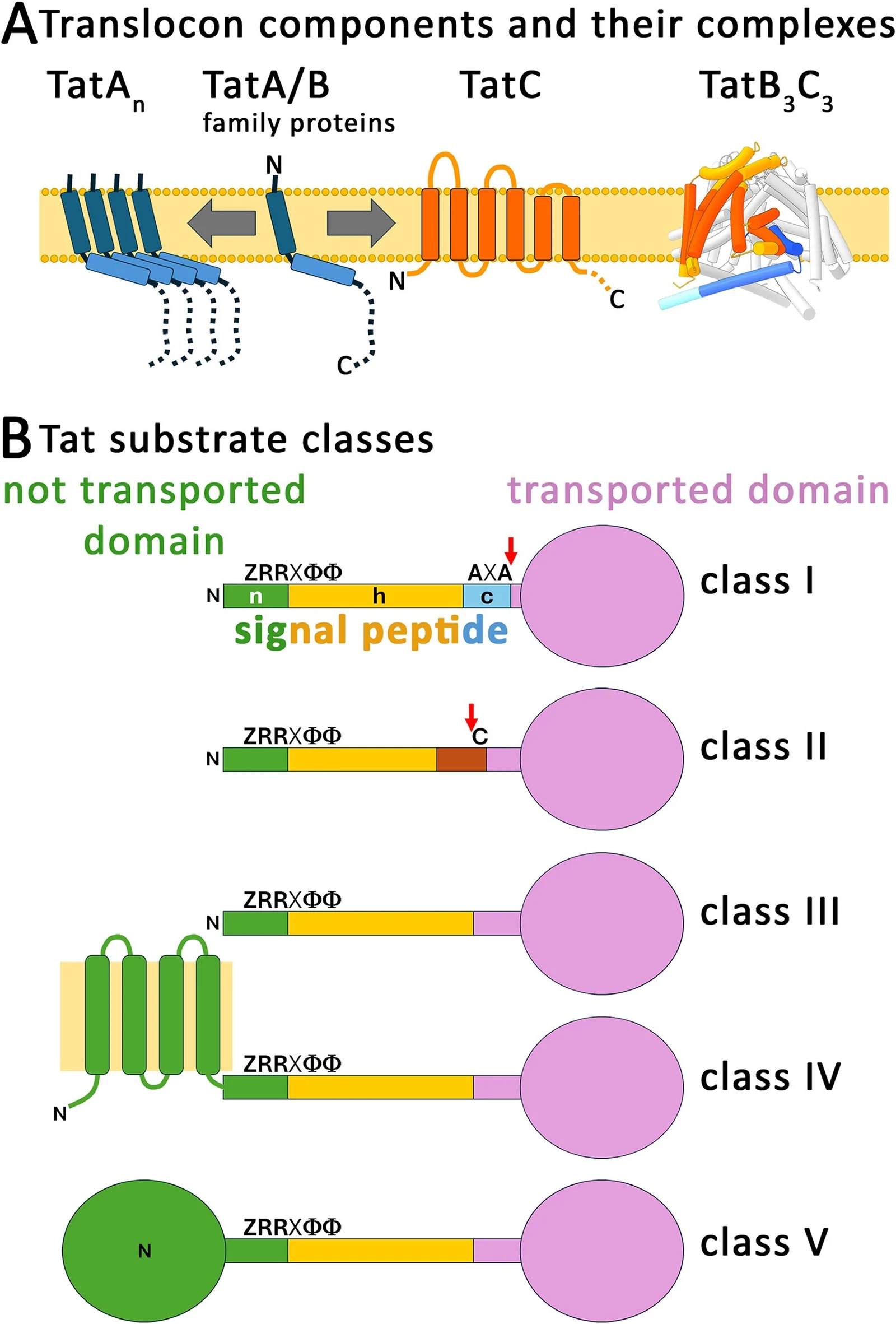
**Tat translocon components and Tat substrate classes. (A)** TatA/B family proteins either assemble to complexes without integrated TatC (left), or they form a stable complex with TatC, such as the illustrated TatB_3_C_3_ complex. Color code: blue, TatA/B family protein; dark blue, membrane anchor; light blue, amphipathic helix; orange, TatC; and dark orange, transmembrane helices of TatC; only one TatC and one TatB in the shown complex is colored. The complex is based on PDB 9DZZ (Deme et al. 2026) and drawn by use of ChimeraX (Goddard et al. 2018). Dotted lines: non-essential C-terminal regions (Behrendt et al. 2004, Lee et al. 2002). **(B)** Tat substrates can be classified according to specific characteristics of the not transported domain and the signal peptide. n-, h-, and c-regions of signal peptides are indicated; Color code: green, not transported domain; yellow, h-region of a signal peptide; blue, c-region of signal peptide; brown, lipobox; and pink, transported domain. The positions of RR-motifs are indicated, as are the cleavage sites of class I and class II signal peptidases (red arrows). See text for references regarding the indicated classes.

Proteins of the TatA/B family are found in two different complexes: they either form a transient homooligomeric complex that can associate with TatC, or they form a stable complex with TatC (Frain et al. 2019) (Fig. 1A). The transient complex is formed out of and disassembles into highly mobile monomers or small oligomers that are distributed in the membrane (Berthelmann and Brüser 2004, Leeuw et al. 2001, Varadarajan et al. 2020). *Three-component Tat systems* possess distinct TatA/B-family proteins for the two complexes, then called TatA and TatB, respectively, which is why these systems are also termed “TatABC systems” (Natale et al. 2008). In *two-component Tat systems* (also called “minimal Tat systems” or “TatAC systems”), the same TatA can fulfil both functions, and is therefore termed “bifunctional TatA” (Jongbloed et al. 2006). *Bacillus subtilis* is the model organism for the analysis of *two-component Tat systems*. In this Gram-positive bacterium, two separate operon-encoded systems exist, TatA_y_C_y_ and TatA_d_C_d_, that function in parallel for specific substrates (Jongbloed et al. 2000, Jongbloed et al. 2004, Pop et al. 2002). Complementation analyses with overproduced components showed that TatC_d_ tolerated all tested Tat substrates, whereas TatC_y_ did not, and TatA_d_ could function with both TatC paralogs, whereas TatA_y_ functioned only with TatC_y_, pointing to an evolved diversity of TatA, TatC, and Tat substrate compatibility (Eijlander et al. 2009). The situation in *B. subtilis* is even more complex, as a monocistronic gene encodes a third TatA paralog called TatA_c_. TatA_c_ can specifically improve transport of the TatA_y_C_y_-containing system, where it interacts with TatA_y_ (Goosens et al. 2015). A TatA_d_C_d_-harboring complex has been detected as a diffuse ca. 230 kDa band in blue native polyacrylamide gel electrophoresis (BN-PAGE) analyses, suggesting that in this case the expected TatAC complex is indeed formed (Barnett et al. 2008). In case of the TatA_y_C_y_ system, a co-purification was demonstrated, which may be due to TatA_y_ that is either laterally associated with or incorporated into TatC_y_-containing complexes (Barnett et al. 2009). Considering the yet limited information about TatC-harboring complexes, especially in phylogenetically distant organisms, it cannot be excluded that functional TatC complexes exist that lack inside integrated TatA/B family proteins. The exact purpose of TatA/B family proteins in TatC complexes has not yet been clearly defined. However, it has been shown with model *three-component Tat systems* that the TatB function is essential, and in fact the first mutants in plant and bacterial systems that abolished Tat transport turned out to be in TatB (Bogsch et al. 1998, Settles et al. 1997, Weiner et al. 1998).

### The five classes of Tat substrates

As mentioned above, TatC-containing complexes of *two*- or *three-component Tat systems* recognize specific signal peptides of the transported Tat substrates (Deme et al. 2026, Gerard and Cline 2006, Zhao and Sazanov 2026). Such signal peptides minimally consist of a hydrophilic n-region with a positive net-charge that is followed by a hydrophobic h-region, with a "twin-arginine" (RR) motif at the interface of the n- and h-regions. In bacteria, the RR-motif was first recognized by the Voordouw group in hydrogenase sequence alignments (Niviere et al. 1992). In case of plant thylakoids, where the pathway was originally termed ΔpH pathway as it was initially believed to be exclusively energized by the ΔpH (Braun et al. 2007, Cline et al. 1992), the RR-motif was at first identified by the groups of Klösgen and Robinson (Chaddock et al. 1995). With a more diverse set of bacterial redox proteins, Berks described thereafter the bacterial RR-motif consensus pattern S/T-R-R-x-F-L-K and proposed that the thylakoid ΔpH pathway is orthologous to the bacterial pathway for redox protein translocation (Berks 1996). When non-redox Tat substrates were included, the more universal RR-motif was defined to have the consensus pattern “Z-R-R-X-Φ-Φ”, in which “Z” denotes a polar or charged residue that is followed by two highly conserved arginines, a non-conserved position (X), and two hydrophobic residues (Φ) (Natale et al. 2008, Yuan et al. 2010). This is the general sequence motif that is recognized by TatC-containing complexes of Tat systems that have been analyzed so far, including archaeal, bacterial, and plant organelle systems (Hou and Brüser 2011). This indicates that this motif evolved very early, before the separation of archaeal and bacterial lineages. If signal peptides are cleavable, they are often cleaved at an “AXA”-like recognition motif that specifies a cleavage site for a type I signal peptidase in a C-terminal region of the signal peptide (c-region) (von Heijne 1983, Lüke et al. 2009). Alternatively, signal peptides can be cleaved by a type II signal peptidase at a lipid-modified cysteine in a lipobox near the end of the h-region (Giménez et al. 2007, Widdick et al. 2011).

Based on the position of their folded domains, and the optional cleavage of their signal peptides, five classes of Tat substrates can be distinguished (Fig. 1B): “Class I Tat substrates” have N-terminal signal peptides that are cleaved by type I signal peptidases, as found in most known cases (Lüke et al. 2009), “Class II Tat substrates” are lipoproteins with N-terminal signal peptides that are cleaved by type II signal peptidases (Shruthi et al. 2010), “Class III Tat substrates” have N-terminal signal peptides that are not cleaved, as found for example in N-terminally membrane-anchored Rieske proteins (Bachmann et al. 2006, Buck et al. 2007, Goosens et al. 2014, Molik et al. 2001), “Class IV Tat substrates” have an internal non-cleaved Tat signal peptide for the transport of C-terminal domains of already membrane-inserted polytopic membrane proteins, as found in Rieske proteins of streptomycetes (Hopkins et al. 2014, Tooke et al. 2017), and “Class V Tat substrates” have an internal non-cleaved Tat signal peptide that is preceded by an N-terminal folded globular domain on the cis-side and enables transport of a C-terminal folded domain to the trans-side, as found in the ferric citrate regulator FecR (Passmore et al. 2020). Some translocated Tat substrates can be integrated into membrane protein complexes, and even with cleaved signal peptides they can remain membrane-attached by interaction with at least one integral membrane protein and/or by a C-terminal membrane anchor (Gallego-Parrilla et al. 2024, Giménez et al. 2007, Hatzixanthis et al. 2003). A Tat substrate that associates with the cell wall has also been identified (Monteferrante et al. 2012).

### Structural aspects of TatC-containing complexes

Recently, CryoEM structures of several TatBC complexes with and without bound signal peptides have been solved (Deme et al. 2026, Zhao and Sazanov 2026). These structures were surprising, as the TatBC components do not form the more packed bundles of TM helices that are usually seen in oligomeric membrane protein complexes. Instead, three TatC subunits assemble together with three TatB to an inverted cup-like structure that is open towards the cis-side and closed at the trans-side (Fig. 2A). The amphipathic helices of TatB formed a triangle-shaped structure on the open surface of the cup-shaped TatC structure. The TatC TM helices TM5 and TM6, together with the membrane-anchors of the TatB subunits, were highly tilted and therefore buried inside the complex. Binding of Tat substrates had only subtle effect on the overall structure of the complexes. The twin-arginine motif was seen to interact with TatC while the signal peptide was sandwiched between a TM helix of one TatB and an APH of another TatB (Deme et al. 2026, Zhao and Sazanov 2026). In one of these studies, a dimeric Tat substrate was found to be bound and translocated by only one of the two signal peptides, which solved the long-standing open question in Tat transport whether or not only one signal peptide of homooligomers is required for transport (Deme et al. 2026).

**Figure 2:**
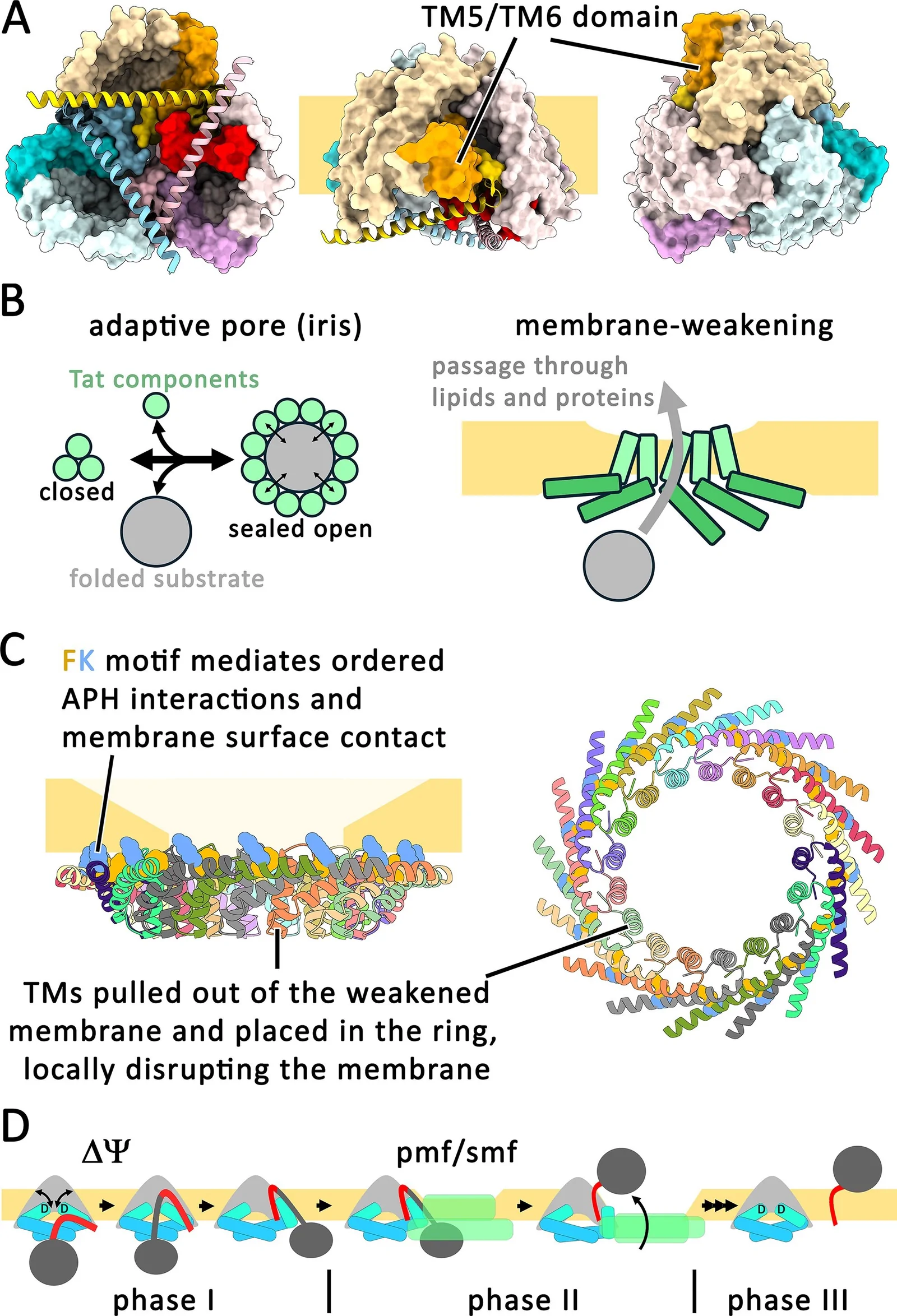
**Tat system complexes. (A)** Structure of the TatB_3_C_3_ complex with one bound signal peptide, based on PDB 9E01 (Deme et al. 2026). Shown are three views of the complex: the bottom (left), the side (middle), and the top (right). The position of the TatC TM5/TM6 domain is indicated and highlighted by more intensive colors of the TatC subunits. Each amphipathic helix of the three TatB subunits is drawn as a ribbon for a better view into the TatC cavity. Color code: turquoise, ochre, and light pink: TatC subunits; blue, yellow, and salmon pink: TatB subunits; and red: Tat signal peptide (of MdoD). **(B)** The principles of previously favored “iris” and “membrane-weakening” mechanisms for membrane permeabilization by TatA. Note that both attempt to explain membrane-sealing during translocation, but neither is able to explain the translocation of linker- or high-pI-Tat substrates, which demands a transient membrane disruption and depolarization. **(C)** AF3 model of the TatA ring that is formed by staggered amphipathic helices, as recently postulated (Brüser and Sanders 2026). Shown is the model of the *Thermoplasma volcanium* TatA_1-49_ ring with 17 subunits (each colored differently), which exemplifies the general ring structure. The positions of the FK motif residues are indicated, as are the TatA membrane anchors that align the pore. **(D)** The postulated translocation process. Phase I: signal peptide (red) binding and insertion at TatC (grey), accompanied with unfolding of the N-terminal region of the mature substrate domain (dark grey) under use of the moving TM5/TM6 domain (turquoise, with “D211” highlighted). The amphipathic helix of TatA/B family proteins is also shown (blue). Phase II: TatA ring docking (transparent green) to the exposed TM5 and membrane anchor extraction, resulting in transient membrane disruption that allows transport (arrow). Phase III: closure of the membrane disruption, release and disassembly of TatA rings, release of the substrate, reset of the TatC-containing complex and recovery of membrane energization. ΔΨ: membrane potential; pmf: proton motive force; and smf: sodium motive force (in halophilic archaea).

In the articles that reported the first TatBC complex structures, either a lateral passage through a TatA assembly or a central passage through TatC were favored (Deme et al. 2026, Zhao and Sazanov 2026). Since a central passage is not consistent with available biochemical data and since, due to a tightly folded periplasmic domain, the structure of *Myxococcus xanthus* TatBC complex argues against a central opening, it was concluded that transport rather occurs through laterally recruited TatA (Brüser and Sanders 2026, Deme et al. 2026). There are strong arguments for a structural symmetry in resting Tat complexes, although an intrinsic asymmetry with one TatB being possibly substituted by a TatA has been proposed in one of the structure reports (Brüser and Sanders 2026, Deme et al. 2026). In haloarchaea, however, two very different TatC-types are required, and it was suggested that in this exceptional case asymmetric complexes are indeed generated (Brüser and Sanders 2026), which will be discussed later in this review.

### Roles of oligomeric TatA assemblies

The role of TatA in membrane permeabilization for the passage of Tat substrates is based on manyfold evidence: (i) TatA is recruited to TatBC complexes to achieve functional translocation (Cline and Mori 2001), (ii) with partially unfolded substrates, translocation arrest occurred in immediate vicinity of TatA (Panahandeh et al. 2008), (iii) Tat substrates could be cross-linked to TatA during active transport (Pal et al. 2013), (iv) TatA can form large homooligomers (Barnett et al. 2009, Beck et al. 2013, Berthelmann et al. 2008, Dabney-Smith and Cline 2009, Gohlke et al. 2005, Leake et al. 2008, Oates et al. 2005), and (v) TatA assemblies can destabilize biological membranes, which has been called “membrane weakening” (Brüser and Sanders 2003, Hou et al. 2018, Mehner-Breitfeld et al. 2022, Rodriguez et al. 2013).

In *E. coli*, analyses of *lacZ* fusions at *tatA, tatB* or *tatC* positions in the respective operon suggested a 50:2:1 TatA:TatB:TatC ratio (Jack et al. 2001), while quantitative immunoblots were reported to indicate a ca. 20:1 TatA:TatB ratio (Sargent et al. 2001). YFP-fused TatA formed complexes of on average 25 TatA, though with a wide range of sizes (Leake et al. 2008), and metallothionein-tagged TatA generated at wild type level clusters of in average 17 ± 6 TatA subunits (Mehner-Breitfeld et al. 2022). In plant plastids (pea), TatA homooligomers of at least 16 protomers assembled (Dabney-Smith and Cline 2009), and an updated determination provided a 21:4:1 TatA:TatB:TatC stoichiometry (Celedon and Cline 2012). The molar excess of TatA over Tat substrates has been questioned by one study that determined a K_M_ for TatA functionality that resembled the K_M_ for a Tat substrate, but K_M_ values do not relate to stoichiometry, and, apart from that, this kinetic analysis treated TatA like a soluble coenzyme, which did not take the membrane integration and complex formations into account (Hauer et al. 2013). Taken together, the published data support the view that TatA is present in excess relative to the other Tat system components, which is expected for a membrane-permeabilizing function of TatA (Cline 2015).

TatA has been demonstrated to interact with Tat substrates already in TatBC-deficient membranes of an *E. coli* mutant strain (Hou et al. 2018, Taubert et al. 2015). This points to potential functionalities in substrate-targeting (Brüser and Sanders 2026). Accordingly, molecular dynamics simulations indicated that membrane-interacting Tat substrates can associate with TatA, and that Tat substrates can stabilize TatA assemblies (Taubert et al. 2015, Tran et al. 2026). In agreement with this view, evenly distributed TatA/fluorescent protein fusions move to TatBC complexes under conditions of recombinant substrate production (Alcock et al. 2013, Rose et al. 2013). A substrate-targeting role would explain why TatA does not remain close to TatBC complexes, which are near the cell poles (Berthelmann et al. 2008, Rose et al. 2013). A targeting role for TatA has been described in early Tat research also for *B. subtilis* and *Streptomyces lividans*, which can form soluble TatA complexes that may target substrates to the translocation site in the membrane (Keersmaeker, van Mellaert, Lammertyn et al. 2005, Keersmaeker, van Mellaert, Schaerlaekens et al. 2005, Schreiber et al. 2006, Westermann et al. 2006).

If there are several TatA paralogs in an organism with *three-component Tat systems*, as often found in Pseudomonadota (the former Proteobacteria), they are sometimes termed TatE if they are not encoded in an operon together with *tatBC* (Sargent et al. 1998). As TatE can functionally replace TatA, it can be regarded as a second TatA paralog (Sargent et al. 1998, Yahr and Wickner 2001). In *E. coli*, TatA and TatE appear to have some preference for specific substrates (Sargent et al. 1998), and the *tatE* gene is induced under biofilm conditions (Beloin et al. 2004), supporting the idea that TatE is a TatA paralog that is optimized for specific substrates and therein resembles TatA_d_ and TatA_y_ of *Bacillus* species (Goosens et al. 2015). It has not yet been analyzed whether this preference is related to targeting, translocation or both potential TatA functions.

### Proposed structures of TatA assemblies

Several structures have been postulated to be formed by TatA alone or in conjunction with TatC-containing complexes, which always had a design that was thought to permit the passage of folded proteins with only a minimal ion leakage at membranes. Distinct mechanisms have been proposed to permeabilize membranes for Tat transport, which can be assigned to one of the following types: (i) “gated pore mechanisms” hypothesized that Tat components enclose substrates inside a translocon with regulated openings, (ii) “iris mechanisms” postulated that Tat components can assemble to a pore able to adapt in its size to substrate dimensions during translocation, (iii) “trapdoor mechanisms” considered transient pores due to movements of TatA domains into the membrane in response to direct substrate contact, and (iv) “membrane weakening mechanisms” suggested that translocation occurs through a locally highly destabilized membrane spot. All proposed mechanisms were initially based on theoretical considerations and knowledge of the component sequences, and were later supported or falsified by structural or biochemical data, as outlined below.

In an early model of the year 2000, termed “sea anemone model”, the groups of Berks and Palmer proposed that TatA forms rings that adapt to the changing diameter of the folded substrate during transport in order to prevent proton leakage (Berks et al. 2000). This adaptation of ring size has been suggested to be achieved either by a “sliding” of individual subunits against each other (“iris mechanism”), or by exchanges of individual subunits during transport (Berks et al. 2000). In the same year, it was also speculated that TatA and TatB might associate with TatC in a way that permits a APH-mediated pushing of substrates through a flexible hole generated by all three components (Wu et al. 2000), and others hypothesized that a gated pore mechanism might be used, without yet attributing functions to the specific Tat components at that time (Musser and Theg 2000). To better explain the driving force for transport and its tolerance to a wide range of substrates, we suggested a “membrane-weakening and pulling model” in 2003, based on the idea that associations of multiple TatA protomers can destabilize membranes, which could permit a TatC-mediated pulling through these destabilized membrane regions (Brüser and Sanders 2003). In 2005, Berks and Palmer suggested a pore that is gated by a “lid” formed by TatA C-termini (Gohlke et al. 2005). In 2013, Berks modified this TatA pore to a star-shaped structure of lidless TatA rings in which the TatA amphipathic helices and C-termini are radially directed outwards, which was an adaptation to our “membrane-weakening and pulling model” (Brüser and Sanders 2003, Rodriguez et al. 2013). In 2018, Hamsanathan and Musser considered three possible variations for an “iris mechanism”, which they termed “symmetric”, “asymmetric”, and “de novo” iris models. In these models, TatA either symmetrically or asymmetrically intercalated between TatC subunits to permit a flexible iris mechanism, or it associated laterally to generate a largely independent iris *de novo* (Hamsanathan and Musser 2018). A symmetric iris mechanism was also suggested as one of the mechanistic options in one of the recent studies on the TatBC-complex (Zhao and Sazanov 2026). However, the symmetric iris mechanisms involve central openings of TatC-containing complexes, which is unlikely to be consistent with the recently published complex structures, especially the one of *Myxococcus xanthus* (Deme et al. 2026). Based on interaction analyses, a similar model proposed an opening of TatC-containing complexes to permit the entry of TatA into a central cavity where translocation takes place (Blümmel et al. 2015). It was furthermore postulated that TatA pore structures can be formed by a trapdoor-like insertion of TatA amphipathic helices into the membrane, which could be stabilized by charge zipper interactions. However, accessibility data and *in vivo* evidence based on site-directed amino acid exchanges later argued against this model (Alcock et al. 2017, Walther et al. 2013). Another trapdoor model suggested that Tat substrates push TM helices of TatA assemblies to some extent deeper into the membrane without changing the TatA topology (Pal et al. 2013). Although the attempt to explain the reversibility of the structural TatA changes during transport was attractive, this model was not consistent with the observation that TatA hinge regions are more exposed upon substrate interactions (Aldridge et al. 2014, Hou et al. 2018).

Currently, “iris” and “membrane weakening” mechanisms, or combinations thereof, are regarded by most research groups as a preferred explanation for membrane permeabilization by TatA (Fig. 2B) (Asher and Theg 2021, Cline 2015, Hamsanathan and Musser 2018, Hao et al. 2022, Palmer and Berks 2024). Based on stimulatory effects of membrane-thinning cell-penetrating peptides, the group of Steven Theg pointed out that membrane thinning at translocation sites might destabilize membranes to an extent that transient toroidal pores could be formed during translocation (McNeilage et al. 2023). Importantly, transport of substrates with a long flexible linker between the signal peptide and the globular mature domain likely demands a transient membrane disruption that permits the passage of the protein through this pore (Cline and McCaffery 2007, Lindenstrauss and Brüser 2009). Similarly, the transport of proteins with a very high pI (such as the model Tat substrate AmiA in *E. coli*) likely demands a transient membrane depolarization, as otherwise transport would be inhibited by a standard membrane potential. We therefore recently extended the “membrane-weakening” model by adding a controlled local and transient membrane disruption step (Brüser and Sanders 2026). Such a pore could be achieved by a conformational transition of an already membrane-weakening TatA ring, and this transition is proposed to be induced by the interaction of this ring with a substrate-bound TatC-containing complex (Brüser and Sanders 2026). Notably, AlphaFold3 (AF3) predicts meaningful TatA ring structures (Abramson et al. 2024, Brüser and Sanders 2026) (Fig. 2C). In these structures, the amphipathic helices align in a staggered and tilted way that agrees with biophysical studies (Walther et al. 2010). The highly conserved “FK” motif near the end of the APH would play a key role in building these structures, which would explain the observed assembly defects of the inactive *E. coli* TatA(F39A) variant (Barrett et al. 2005, Hou and Brüser 2011). The ring structure as predicted by AF3 would be actually generated by uniformly interacting staggered amphipathic helices. The highly conserved “FK” motif was interpreted as evidence for a key role of such a TatA ring structure in Tat-dependent transport (Brüser and Sanders 2026). Despite explaining previously puzzling findings, the AF3-predicted TatA ring structure certainly requires experimental validation.

The conservation of the FK motif in all TatA paralogs may thus be indicative for the ability to form mixed complexes, such as the reported TatA/TatE complex in *E. coli*, or TatA_y_/TatA_c_-complex in *B. subtilis* (Eimer et al. 2015, Goosens et al. 2015). In plastids and cyanobacteria, TatA paralogs have a conserved FQ motif instead of the FK motif in their APH (Brüser and Sanders 2026). As the “K” of the FK motif is proposed to mediate phospholipid interactions, the “Q” of the FQ motif in cyanobacteria and plastids could represent an adaptation to the abundant galactolipids in these cases (Brüser and Sanders 2026). Interestingly, plant TatB (Hcf106) and TatA (Tha4) have both this FQ motif, which implies that they may be able to form mixed complexes. This would mean that “TatB” (Hcf106) of plant plastids could be a bifunctional TatA rather than a TatB that only interacts with TatC. As TatA (Tha4) is essential for Tat transport in plastids, mixed Tha4/Hcf106 rings could be essential for plant plastid systems (Jessup et al. 2026). The additional function of TatB (Hcf106) in plastids would rationalize that the TatB:TatC ratio in plants has been determined to be ca. 4:1 (Celedon and Cline 2012), and it would also explain that up to eight TatB subunits can be self-crosslinked via disulfide bridges in complexes that are not part of TatBC complexes (Ma et al. 2018). *E. coli* TatB has no FK motif (“LR” is at this position), suggesting that this TatB is not as stably integrated into TatA rings and more specialized for TatC interactions. Notably, when the *tatABC* genes are overexpressed, traces of TatB could be co-purified with TatA, but BN-PAGE analyses indicated that these associations were small and thus likely not able to form large and stable mixed TatA/TatB rings (Gohlke et al. 2005, Oates et al. 2005).

### The molecular mechanistic model for Tat transport

A first molecular model for Tat transport has recently been proposed that takes the predicted TatA ring, the known TatB_3_C_3_ complex structure, and available biochemical data into account (Brüser and Sanders 2026) (Fig. 2D). This model is in full agreement with the well-established view that Tat signal peptides bind to TatC-containing complexes, and that the Tat substrates are then translocated through a laterally associated TatA assembly. It further integrates the TatC-dependent signal peptide insertase activity as an early step in translocation, as previously suggested by various groups (Brüser and Sanders 2003, Fröbel et al. 2012, Ramasamy et al. 2013), and the TatA-dependent membrane weakening, as favored by the majority of the currently active Tat research groups (Deme et al. 2026, McNeilage et al. 2023, Mehner-Breitfeld et al. 2022, Palmer and Berks 2024).

The model postulates a signal peptide insertion function by the TatC-containing complex as initial step of Tat transport (phase I), followed by a TatA-dependent local membrane disruption and depolarization for the passage of the folded region of the protein (phase II) and a substrate-release, TatA ring disassembly, TatBC complex reset, and local membrane potential recovery (phase III) (Brüser and Sanders 2026). In contrast to the predecessor models that all assumed a transport designed to prevent membrane depolarization, this new molecular model postulates that the membrane needs to be transiently and locally disrupted and depolarized. Key arguments for this model are (i) flexible linkers between the signal peptide and the folded mature domain are tolerated by Tat systems (Cline and McCaffery 2007, Lindenstrauss and Brüser 2009), (ii) translocation-incompatible exposed hydrophobic regions (such as unfolded globular proteins) interrupt transport without blocking the translocon (Cline and McCaffery 2007, Richter and Brüser 2005), and (iii) “high pI”-Tat substrates with a positive net charge, such as AmiA, can still be translocated (Ize et al. 2003). If the translocation efficiency of heterologous Tat substrates is low due to used linkers or exposed hydrophobic regions that can be trapped by interactions with the disrupted membrane, the model predicts that translocation is interrupted without blocking the translocon, as the inserted signal peptide can be released into the membrane where it can be cleaved by a signal peptidase, which is supported experimentally (Cline and McCaffery 2007, Di Cola and Robinson 2005, Richter and Brüser 2005, Richter et al. 2007). This would explain the specific properties that had been termed “folding quality control”, which in principle can be used for biotechnological production of correctly folded functional pharmaceutical proteins, as shown in many proof-of-concept studies (DeLisa et al. 2003, Fisher and DeLisa 2004, Fisher et al. 2006, Fisher et al. 2011, Sutherland et al. 2018). C-terminal TatB domains have chaperone-like activities, and mutations in that region can affect the transport efficiency of not fully folded globular proteins (Taw et al. 2022). In other studies, it has been observed that Tat signal peptide-fused not fully folded proteins and natively unfolded proteins with exposed hydrophobic regions terminated translocation at a late stage, resulting in growth defects that were not prevented by TatB (Richter and Brüser 2005, Richter et al. 2007). Furthermore, if there are no exposed hydrophobic stretches, small unfolded Tat substrates or Tat substrates with long unstructured linkers are tolerated by Tat systems, suggesting that only exposed hydrophobic regions are problematic for Tat transport, and globular structures are not needed (Cline and McCaffery 2007, Lindenstrauss and Brüser 2009, Richter et al. 2007).

The mechanistic model postulates that the observed signal peptide insertase activity is a major function of TatC (Brüser and Sanders 2026, Fröbel et al. 2012). More specifically, it suggests that a ΔΨ-energized movement of the TM5/TM6 domain within TatC is required for the signal peptide insertion (Fig. 2A and D). This conformational change of the TatC TM5/TM6 domain is proposed to be facilitated by a conserved charge (in *E. coli* D211; from now on dubbed in any TatC “D211”) (Bageshwar and Musser 2007). Although “D211” is highly conserved in TatCs and is classified as essential (Buchanan et al. 2002), some mutations of this residue in *E. coli* have residual activity that can complement transport with certain substrates, especially with multicopy suppression (Holzapfel et al. 2007). Therefore, there might be a substrate-specific essential role of a ΔΨ-energized TM5/TM6 movement, which can be the N-terminal unfolding during signal peptide insertion, while the TM5/TM6 domain surface exposure could also be achieved by the signal peptide insertion alone (Brüser and Sanders 2026, Holzapfel et al. 2007), but these ideas are not yet addressed experimentally.

The model suggests a mechanism for a controlled and transient local membrane disruption, based on membrane-weakening and easily extractable N-terminal TatA membrane anchors (Hao et al. 2022, Hou et al. 2018, Mehner-Breitfeld et al. 2021, Stockwald et al. 2022). When docking to the TatC TM5/TM6 domain, the N-terminal TatA TM anchors are proposed to be extracted from the membrane by a concerted conformational transition of the interacting APHs, resulting in a controlled local membrane disruption through which the substrate then passes the membrane (Brüser and Sanders 2026). According to this model, the directionality and efficiency is proposed to result from the fact that the C-terminal region of the signal peptide is already at the trans-side of the membrane, and that the N-terminal part of the transported domain is partially unfolded during the signal peptide insertion process conducted by TatC. The transient pore would permit a completion of folding and relaxation of the extended protein structure, enabling the brownian-ratchet-driven diffusion of the globular domain toward the c-region of the signal peptide that is already on the trans side. According to this suggested mechanism, linker sequences sandwiched between the signal peptide and the globular domain are expected to be tolerated but reduce the transport efficiency, depending on their lengths, as has been observed experimentally (Cline and McCaffery 2007, Lindenstrauss and Brüser 2009).

It is proposed that the closure of the transient membrane disruption is spontaneous, and that association with the membrane-stabilizing proteins of the PspA family prevents extended membrane damage (Brüser and Sanders 2026). An association of PspA family proteins with Tat system components has been reported for *E. coli* and *B. subtilis* (Bernal-Cabas et al. 2020, Mehner et al. 2012). *E. coli* PspA was found to relieve saturation of Tat transport, which agrees with a function in support of rapid membrane closure and re-energization after transport (DeLisa et al. 2004). In plant plastids, the PspA homologue Vipp1 stimulated Tat transport as well, while a direct Vipp1/Tat interaction could not be demonstrated (Lo and Theg 2012).

### Asymmetric Tat systems in halophilic archaea

In symmetric TatC-containing complexes, such as found in *B. subtilis* or *E. coli*, a local and transient membrane depolarization during Tat transport would require a regulation for a coordinated function of the three equivalent signal peptide binding sites (Alder and Theg 2003, Brüser and Sanders 2026). An alternative to a regulated coordination can be an asymmetry in TatC-containing complexes that results in just one Tat substrate binding site. As single TatCs can only assemble to symmetric complexes with identical binding sites for TatA/B family proteins and Tat substrates, we searched for Tat systems with two or more TatC components. Among bacteria, strains with two TatCs often occur in Chloroflexota and Bacillota, but all these TatCs possess the typical conserved residues of the RR-motif binding site, indicating normal functionality. They thus can be expected to be part of separate Tat systems that can function in parallel, as known for *B. subtilis* (Jongbloed et al. 2004). However, already >20 years ago, it has been recognized that halophilic archaea such as *Haloferax mediterranei* or *Halobacterium salinarium* possess an unusual TatC with 14 TMs (Bolhuis 2002, Dilks et al. 2005, Hutcheon and Bolhuis 2003, Yen et al. 2002). In all of these initial descriptions, it was recognized that the 14 TM-TatCs represented two fused TatCs, connected by a domain with two additional TM helices (Fig. 3A). Next to the gene of the 14 TM-TatC was a gene encoding a second TatC with a usual 6 TM topology. The Tat system of haloarchaea is responsible for the large majority of secreted proteins, which is unique and proposed to relate to effects on folding by the high intracytoplasmic concentration of potassium in these prokaryotes (Pohlschröder et al. 2005, Rose et al. 2002). The Pohlschröder-group was the first that showed in a mutational analysis that in *H. mediterranei* both *tatC* genes were essential for this organism, as was one of its two *tatA* genes. They termed the long 14 TM TatC “TatC_t_” and the genetically linked 6 TM TatC “TatC_o_” (Dilks et al. 2005). In the same study, they also recognized that in *Haloarcula* species the 6 TM TatC found in other halophilic archaea had a C-terminal extension of 4 TMs, resulting in a 10 TM TatC. Later, the TatC proteins of halophilic archaea were classified into three combinatory groups, in all of which a 14 TM TatC_t_ is combined with either a 6 TM TatC_o_ (group I), a 6 TM TatC with an N-terminal extension (TatC_n_) (group II), or a 10 TM TatC_x_, in which a 6 TM TatC is C-terminally extended by 4 TMs (group III) (Ghosh et al. 2019). It was proposed by Thomas and Bolhuis that the two different TatCs form a functional unit (Thomas and Bolhuis 2006).

**Figure 3:**
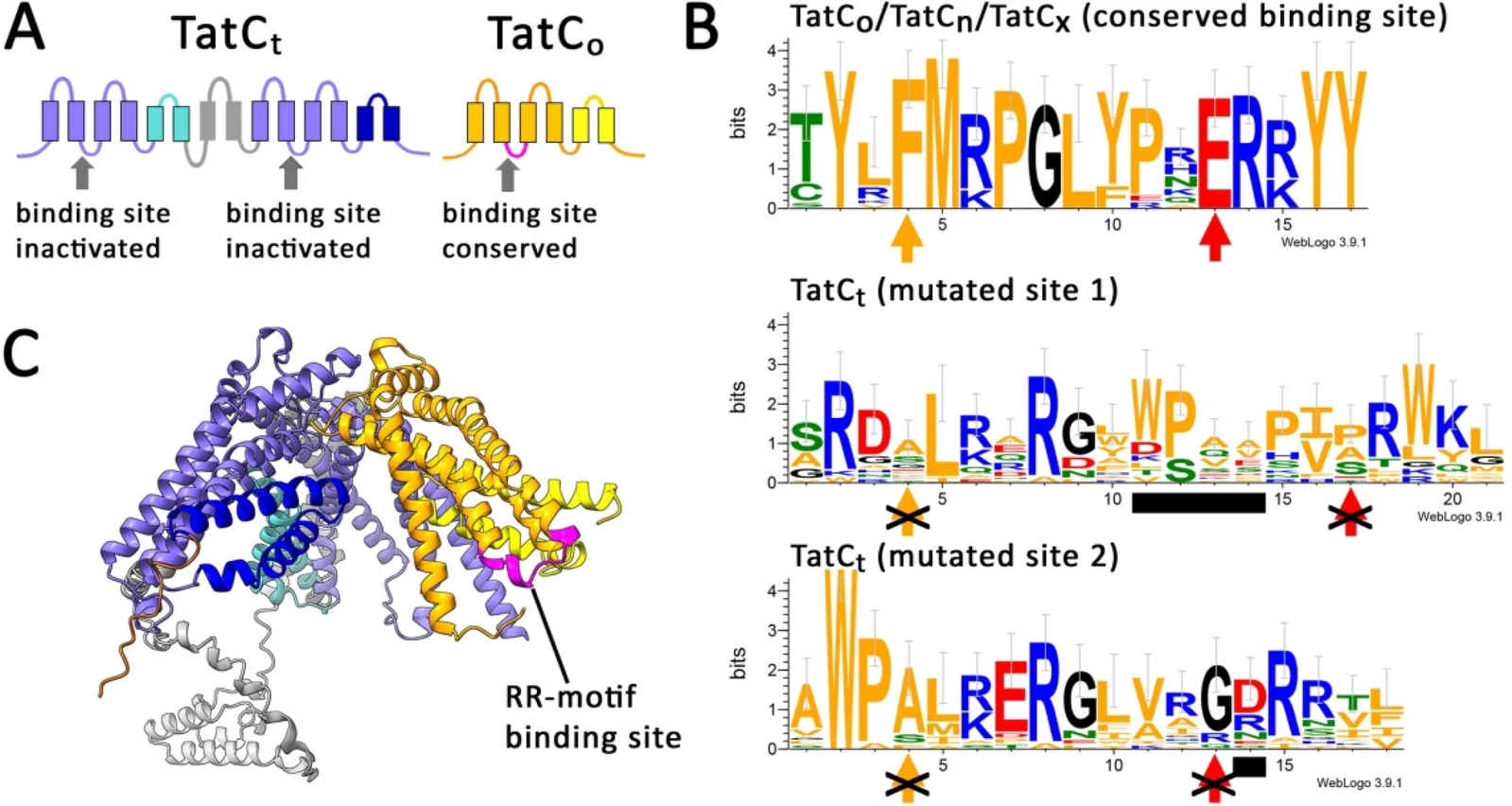
**Asymmetric TatC-containing complexes in haloarchaea. (A)** Topology of TatC_t_ and TatC_o_ of *Halobacterium*. TMs of TatC_t_ that correspond to TM 1–4 of 6TM TatCs are in purple, TMs that correspond to TM5 and TM6 of 6TM TatCs are in light and dark blue. TMs of TatC_o_ that correspond to TM 1–4 of 6TM TatCs are in orange, TMs that correspond to TM5 and TM6 of 6TM TatCs are in yellow. The first cytoplasmic loop that contains the functional RR-motif binding site is colored magenta. The positions of the two inactivated and one conserved binding sites are indicated. **(B)** WebLogo (Crooks et al. 2004) plots of the conserved binding site in TatC_o_, TatC_n_, and TatC_x_ (upper plot), and of the mutated binding sites 1 and 2 of TatC_t_ (middle and lower plot, respectively). Color code: hydrophobic residues, orange; polar uncharged residues, green; positively charged residues, blue; negatively charged residues, red; and glycine, black. Black bars: insertions; arrows indicate positions corresponding to F94 and E103 in *E. coli* TatC. Crossed arrows indicate mutated F94/E103 positions. The plots are based on Clustal Omega alignments of sequences from 13 genera of haloarchaea (*Haloarcula, Halobacterium, Haloferax, Halogeometricum, Halomicrobium, Haloquadratum, Halorhabdus, Halorubrum, Haloterrigena, Halovivax, Natrialba, Natronobacterium*, and *Natronomonas*) (Sievers et al. 2011). **(C)** Structure of the heterodimeric TatC_t_/TatC_o_ complex of *Halobacterium salinarium*, as predicted by AF3, without the potential inclusion of TatAs. Color code as in **(A)**.

Early sequence-based analyses of functional divergence already suggested for TatC_t_ and TatC_o_ a “subfunctionalization after gene duplication, in which the sum of functions of the two paralogs complements the ancestral function” (Jiang and Fares 2011). A closer look at the 14 TM TatC_t_ indicates that the RR-motif binding sites of the two “fused” TatC are most likely both inactive, as evidenced by the absence of the otherwise in archaea and bacteria highly conserved F94 and E103 residues (*E. coli* numbering), which are the key residues for the RR-motif binding (Deme et al. 2026) (Fig. 3A and B). In contrast, this RR-motif binding site of the 6 TM TatC_o_ (as in the 10 TM TatC_X_) appears to be functional, as both residues are present, and also their direct environment is highly conserved (Fig. 3A and B). These characteristics strongly suggest that the 14 TM TatC_t_ must interact with a 6 TM TatC_o_ or 10 TM TatC_x_ to form active TatC-containing complexes, which would then have an overall structure similar to the recently published trimeric bacterial TatC-based complexes (Deme et al. 2026, Zhao and Sazanov 2026) (Fig. 3C). Importantly, this fully agrees with the above mentioned prediction by Jiang and Fares (Jiang and Fares 2011).

Why did haloarchaea evolve asymmetric Tat complexes? One reason could be that the extremely high (several molar) salt concentrations in the habitat and inside the cytoplasm of these species interfere with the regulatory mechanism for activity coordination at symmetric Tat complexes, which may rely on salt bridges (Christian and Waltho 1962).

### Protein targeting in early evolution—Is TatC the most ancient translocase?

How did Tat systems evolve? When first membranes appeared in evolution, proteins needed to be targeted into or across membranes to facilitate selective uptake or release of compounds, and to place proteins on the outside when this was advantageous for the evolving cell (Mansy 2010, Schrum et al. 2010). Three types of translocases are known to have ancestors that evolved before archaea and bacteria separated, and all of them can function as insertases of signal peptides: SecY-based systems, YidC, and TatC-based systems (Cao and Saier 2003, Lewis and Hegde 2021, Yen et al. 2002) (Fig. 4A). SecY-family proteins are essential for all known organisms, showing monophyletic and highly divergent evolution in archaea and bacteria (Cao and Saier 2003). SecY associates laterally with more components, such as SecE or SecG, but SecY forms the core of the translocon and its ancestor has been proposed to catalyze membrane protein insertion alone in early evolution (Lewis and Hegde 2021). A chaperone-like system consisting of a signal recognition particle (SRP) and an SRP receptor coevolved with the SecY-based systems to permit a cotranslational targeting that prevents aggregation of hydrophobic TM helices (Harris and Goldman 2021, Saraogi and Shan 2014). SecY evolved from a fused duplication of an ancestor that was also the ancestor of today’s YidC (Lewis and Hegde 2021). All these systems recognize a positively charged region, followed by an insertion of an adjacent hydrophobic region. While YidC can insert a hydrophobic region on either side, Sec systems as well as TatC insert only hydrophobic regions that are on the C-terminal side of the positively charged region (Fig. 4A). YidC thus cooperates with Sec-systems when N-out topologies are generated or when TM helices are released from SecY (Hegde and Keenan 2024, Kuhn and Kiefer 2017). However, YidC is also capable to function independently in membrane protein insertion (Kuhn and Kiefer 2017, Lewis and Hegde 2021).

**Figure 4:**
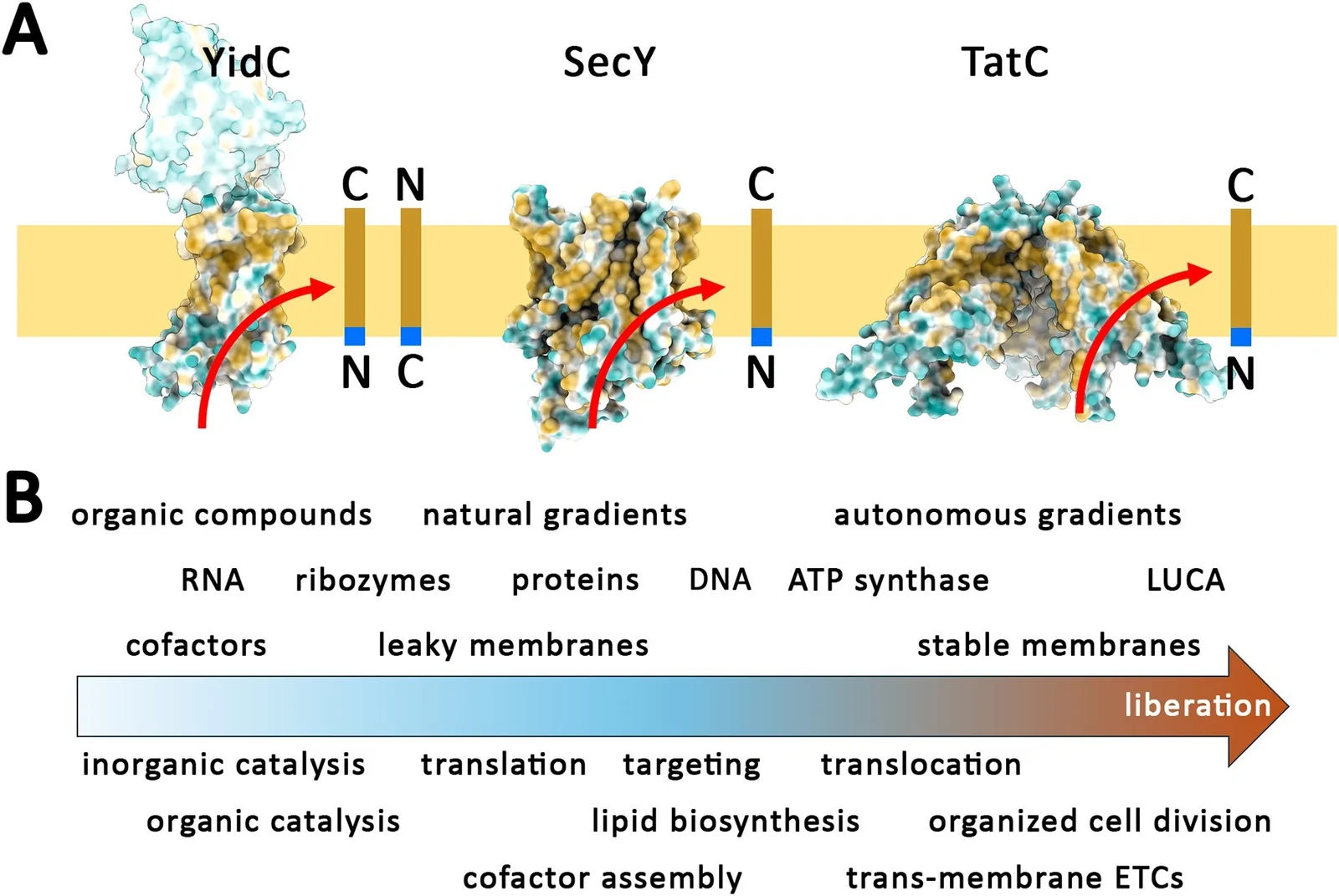
**Placing the origin of TatC within the current understanding of pre-LUCA evolution. (A)** Activities of the ancient signal peptide insertases YidC, SecY, and TatC. The structures show surface characteristics in terms of hydrophobicity (ochre, hydrophobic and turquoise, hydrophilic), generated by ChimeraX (Goddard et al. 2018). The YidC structure is based on the AF3 data base (AF-Q7MQK5-F1-v6), the SecY structure on PDB 1RHZ (van den Berg et al. 2004), and the TatC_3_ complex model was generated by AF3 (based on *E. coli* TatC). The insertion of a hydrophobic region (dark ochre) next to a positively charged region (blue) is catalyzed in the indicated orientations. **(B)** Current hypothetical view of the pre-LUCA evolution, with the evolved components, conditions and processes ordered according to an arbitrary time scale (arrow). DNA may have evolved later (Forterre 2024); Tat systems are associated with “translocation”.

Membrane protein biogenesis is considered the only function of ancient SecY/YidC-based systems, although today’s Sec machineries can translocate proteins across the membrane as well. The ATPase SecA is required for cotranslational or posttranslational translocation of soluble proteins or protein domains in bacteria, and there is no SecA homolog in archaea, indicating a late evolution of Sec-dependent translocation of soluble proteins in bacteria (Neumann-Haefelin et al. 2000, Pohlschröder et al. 2004, Sääf et al. 1995, Zhu et al. 2022). Although an archaeal SecA homolog does not exist, there is evidence for an analogous SecA-like pathway in archaea (Irihimovitch and Eichler 2003). These considerations suggest that the Tat system may have been the first translocation apparatus for soluble proteins in evolution. This assumption makes sense, as processes that permit translocation of unfolded proteins and extracytoplasmic folding first needed to evolve before such a transport was possible. Moreover, many ancient proteins required cofactors for their basic functions, and biogenesis and insertion pathways for these cofactors were located inside the cytoplasm. Since a cytoplasmic cofactor insertion machinery already existed, it could be used for translocated enzymes in case of Tat transport. Until today, one main reason for the use of Tat transport is still a cytoplasmic assembly of cofactors that are required for proteins functional in extracytoplasmic spaces. Such cofactors that have been experimentally demonstrated to be inserted into Tat protein substrates prior to transport include Fe-S-clusters ([4Fe-4S], [2Fe-2S]) (Bachmann et al. 2006, Brüser et al. 2003, Molik et al. 2001), molybdenum cofactors (Mo-bis-MGD, Mo-MCD) (Neumann et al. 2009, Ray et al. 2003), nickel-iron complexes (Rodrigue et al. 1999), flavins (Panasia et al. 2021), coenzyme F_420_ (Bashiri et al. 2012), NADP (Halbig et al. 1999), heme (Miethke et al. 2013, Sturm et al. 2006), and pyridoxal phosphate (Ringel et al. 2016, 2017). In addition, enzymes with cobalt-containing cofactors (norpseudo-B12) that can insert their cofactor inside the cytoplasm, contain Tat signal peptides and are functional outside the cytoplasm (John et al. 2006). However, copper cofactors are exceptions: as copper is highly toxic inside the cytoplasm, Tat-dependently translocated copper proteins fold already without copper assembly in the cytoplasm, and copper is inserted after transport across the cytoplasmic membrane (Stolle et al. 2016).

Based on the current views on early evolution of metabolism and membranes, the appearance of Tat systems can be integrated into the hypothetical sequence of events during pre-LUCA evolution as follows (Fig. 4B): When the RNA-based “ribozyme” world, in which small and simple peptides are believed to have played an assisting role, was extended to an “ribozyme/enzyme” world by the invention of ribozyme-based translation, more and more functions were transferred to or invented by proteins (Fried et al. 2022). Due to the complex biosynthetic requirements of DNA, proteins are anticipated to have preceded DNA, and translation of imprecisely replicated RNA yielded manifold mutated protein sequences that were positively selected when conferring advantageous functions (Forterre 2024, Goldman et al. 2010). When first membranes emerged, which were leaky as they consisted of abiotically formed long chain amphipathic compounds like fatty acids (Chen and Walde 2010, Sojo et al. 2014), hydrophobic peptides could partition into such membranes. This permitted already a selection for those peptides that conferred certain advantages, such as selective permeability of needed compounds, leading to the evolution of the first membrane proteins. The evolution of lipid bilayers and membrane proteins went hand in hand, as only proteins—not RNA—could be stably placed into lipid environments to provide the necessary selective permeabilization of lipid bilayers. When biological fatty acid and phospholipid synthesis pathways evolved, membranes became thicker, tighter and more stable, while the insertion of proteins gradually required more and more assistance by insertases, which became the ancestors of today’s protein insertion and translocation systems that include the SecY, YidC, and TatC components.

How can the origin of Tat systems be imagined? As mentioned above, TatC inserts signal peptides with a N-in/C-out topology into membranes, much like SecY (Fröbel et al. 2012). However, while SecY laterally inserts any TM domains into the membrane (Gumbart and Schulten 2007, Hou et al. 2012, Rapoport et al. 2004), the insertion by TatC stops when an intermediate is formed that is with its signal peptide deeply buried in the complex (Gerard and Cline 2007). Instead of releasing the bound signal peptide into the membrane, which happens in the absence of TatB, TatC of complete Tat systems permits the lateral passage of the globular Tat substrate domain through a TatA-disrupted membrane before catalysis of translocation by Tat is completed by the release of the signal peptide into the membrane (Fröbel et al. 2012). A function of TatB in translocation is therefore the prevention of signal peptide release prior to transport. It is possible that initially, at times of leaky membranes, folded domains could easily flip through the membrane, and thus TatC alone may have had “flippase” activity, rapidly transferring folded proteins from the cis- to the trans-side. However, when membranes became stronger, TatC may have required the assistance of TatA/B family peptides that evolved to transiently disrupt the membranes when interacting with TatC, and TatA/B family proteins became part of all Tat systems from archaea to bacteria and their derived organelles (Yen et al. 2002).

To be translocated by Tat, the existing globular enzymes did not need to be kept in an unfolded state, which was an advantage of the Tat pathway. However, to facilitate and selectively enable the transport by Tat, these globular enzymes acquired early in evolution specific N-terminal transport signals—the highly conserved Tat signal peptides.

### The evolution of signal peptides—from membrane-protein insertion to Tat-transport

According to the “positive-inside-rule” of membrane proteins, the membrane protein regions next to the inner side of the membrane have a positive net charge (von Heijne 1986, 1989). The same rule is true for signal peptides of Sec- or Tat-dependently transported soluble proteins, which have the same targeting function as the first membrane-inserted helices of membrane proteins (Saraogi and Shan 2014, Schibich et al. 2016, Ullers et al. 2006). It is likely that these characteristics originated from the ability to associate with negatively charged lipid head groups via the n-region, and to anchor to these membranes via the h-region (Jones et al. 1990, Keller et al. 1992, Nesmeyanova et al. 1997), which probably facilitated membrane insertion in early evolution.

To be membrane-targeted, Tat substrates just needed such a targeting module at their N-terminus. Still today, Tat substrates can be targeted to the membranes and can be transported thereafter (Bageshwar et al. 2009). However, the signal peptides needed to be differentiated from Sec signal peptides, for that only Tat substrates could be targeted by the Tat system. Today’s Tat-signal peptides have a lower average hydrophobicity, which can influence the specificity (Cristóbal et al. 1999). However, this cannot be the only criterium, as there is significant overlap between the distributions of Sec and Tat signal peptide hydrophobicities. A second criterium could have been the RR-motif in Tat signal peptides. However, when a RR-motif was introduced into a Sec signal peptide, transport of the mutated protein was still Sec-dependent (Brüser et al. 1998). Even when the entire signal peptide of a Sec substrate was exchanged by a Tat signal peptide, transport remained Sec dependent unless the protein was allowed to fold inside the cytoplasm (Sanders et al. 2001). Moreover, complete inactivation of RR-motifs has been shown to still permit a targeting to the Tat translocon (Huang et al. 2017, Ulfig and Freudl 2018). A third criterium could have been a Sec-avoidance motif, which has been defined as positively charged c-region of Tat signal peptides (Bogsch et al. 1997). However, positive charges are only present in a subset of Tat substrate c-regions, and it has been shown that most Tat signal peptides can mediate Sec transport when fused to a Sec substrate (Tullman-Ercek et al. 2007). In summary, it is likely an interplay of mature domain characteristics, signal peptide hydrophobicities, and charge distributions that together determine the targeting pathway to the correct translocon. One important aspect could be that Sec-targeted proteins are either already recognized by SRP or SecA at the ribosome for cotranslational targeting, or they are fully translated and then kept in an unfolded Sec-compatible state by chaperones such as SecB (Driessen 2001, Saraogi and Shan 2014, Zhu et al. 2022). In other words: Sec substrates are never freely inserted into membranes prior to the translocon interaction, whereas Tat substrates can be inserted into the membrane and transported thereafter, which could be the key difference in the targeting pathways (Bageshwar et al. 2009, Shanmugham et al. 2006). Notably, Tat substrates could be bound to purified solubilized TatC-containing complexes, showing that a membrane-independent targeting pathway may exist, but it is not known yet whether such a direct binding of Tat substrates to such complexes occurs *in vivo* with physiological substrate concentrations (Deme et al. 2026).

Many signal peptide-binding chaperones have been identified that were thought to be able to target Tat substrates to their translocon. Several of these chaperones are already genetically linked to specific substrates that contain for example Ni-Fe-centers or molybdenum cofactors (Turner et al. 2004). These are specific chaperones, termed “redox enzyme maturation proteins” (REMPs), that use signal peptides as platforms to support the insertion of the required cofactor into the mature domain of these redox proteins (Genest et al. 2008, Li et al. 2010, Schubert et al. 2007). Early models that suggested a direct targeting function for such chaperones to the Tat system could not be confirmed (Bageshwar et al. 2021, Ray et al. 2003).

In contrast to REMPs, some general chaperones, DnaK and SlyD, have been identified that bind a broader range of Tat signal peptides (Graubner et al. 2007, Oresnik et al. 2001). DnaK is an ATP-dependent chaperone that is highly abundant and involved in folding of many proteins, often in cooperation with other proteins (Deuerling et al. 2003, Deuerling et al. 1999, Hesterkamp and Bukau 1998). SlyD is less abundant, has minimally a prolyl isomerase and chaperone domain, and has been shown to be a folding chaperone (Scholz et al. 2006, Weininger et al. 2009). Some SlyD homologs have a C-terminal nickel-binding domain that is involved in intracellular nickel storage. However, SlyD binds a broad range of Tat signal peptides without preference for nickel-containing Tat substrates (Lindemeier et al. 2024). *E. coli* SlyD bound the Tat signal peptide of the model Tat substrate HiPIP of *Allochromatium vinosum* with high affinity (K_D_ ca. 0.5 µM), which is in the range of substrate-specific REMPs (Lindemeier et al. 2024). The twin-arginines in the Tat signal peptide were shown to contribute to the high affinity (Lindemeier et al. 2024). As SlyD is not involved in cofactor insertion, it might play a role in signal peptide protection or membrane targeting. Interestingly, SlyD is hijacked by phage ΦX174, targets its lysis protein to the membrane and incorporates together with this protein into an cell-wall-biosynthesis inhibitory complex that promotes lysis of infected cells (Orta et al. 2023). The requirement of this host protein for cell lysis by ΦX174 is actually the original reason for its name (sensitivity to lysis: Sly) (Roof and Young 1995).

### Early Tat transport without ΔΨ? Acidophilic archaea show what is possible

So far, this review discussed our knowledge about today’s Tat systems and the evolution of Tat systems in context of the evolution of cytoplasmic membranes, but we did not consider the energetic situation during early evolution. There seems to be a contradiction that on one hand Tat systems evolved in leaky membranes of pre-LUCA times, while on the other hand Tat transport is known to depend on the membrane energization. Today, this membrane energization can be either a pmf or a smf (Bageshwar and Musser 2007, Kwan et al. 2008, Theg et al. 2005). Both have the electric field (ΔΨ) in common, which is postulated to be the driving force for signal peptide insertion by energizing a movement of the TatC-TM5/TM6 domain, to which the residue “D211” in that domain may contribute (Brüser and Sanders 2026).

However, as noted above, early membranes were leaky and are assumed to have required the evolution of fatty acid and phospholipid synthesis to become more sealed (Sojo et al. 2014). According to the current view, the diversification of membrane lipids in archaea and bacteria followed later (Koga 2014, Lombard et al. 2012). It is anticipated that initially natural pH gradients, which were possibly converted to sodium gradients that are more stable at the leaky early membranes, were used to energize ancient membranes (Martin 2011, Mulkidjanian et al. 2007, Sojo et al. 2014). In any case, it is unlikely that a ΔΨ of these membranes was comparable to today’s membranes. Therefore, the basic question remains, whether Tat systems could have functioned in membranes without significant ΔΨ.

A closer look at the phylogenetic tree of TatC sequences in archaea and bacteria helped to answer this question. Saier et al. had already reported such a tree, which was the first evidence for an ancient origin of Tat systems in LUCA (Yen et al. 2002). Our extended tree with exemplary representatives of known phyla with Tat systems shows that indeed TatC of archaea and bacteria are clearly separated and thus diverged early in evolution, supporting the existence of the Tat system in LUCA (Fig. 5). Within the bacteria, TatC sequences clustered—as expected—largely according to phylogenetic relationships of the respective organisms, which was already observed in an earlier study (Simone et al. 2013).

**Figure 5:**
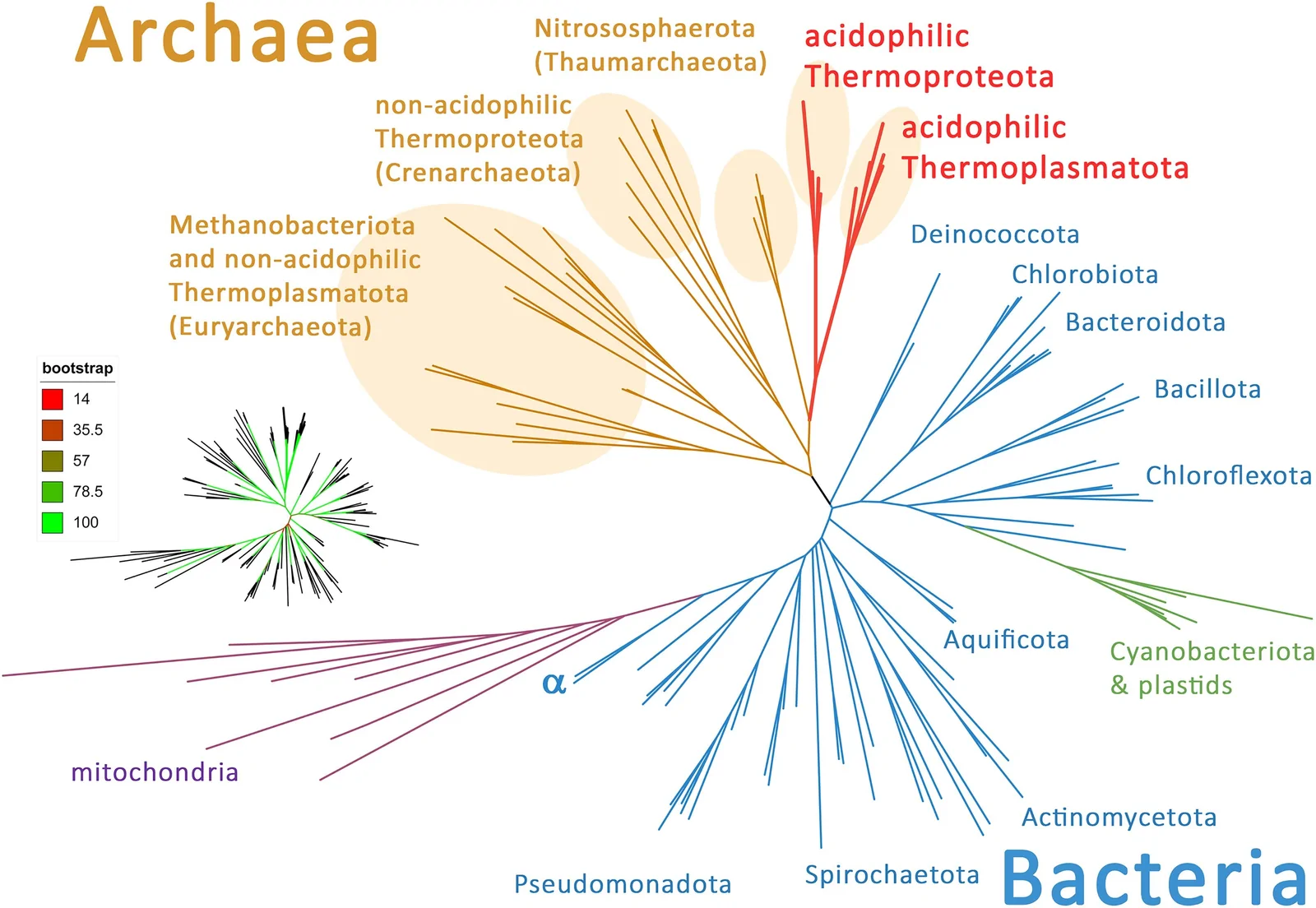
**Adaptation of TatC to acidic habitats dominates over organismic clustering in archaea**. Phylogenetic tree of 108 TatC sequences from representatives of Tat-system-containing bacteria, archaea, plastids and mitochondria, highlighting the separate clades of the extremely acidophilic archaea that do not group according to the organismic phylogeny. For the analysis, the TatC alignment was generated by use of MAFFT7 with Smith-Waterman-based local pairwise alignment to account for the highly divergent sequences with conserved domains, variable N- and C-termini and insertions and deletions. Up to 1000 iterations were allowed to increase accuracy. Trimming was done using CLIPKIT software in the kpic-smart-gap mode. The final tree was generated by IQ-TREE with automated model finder, ultrafast bootstrapping with 1000 replicates, and an SH-aLERT-Test with 1000 replicates. Color code: red, extremely acidophilic archaeal TatCs; ochre, other archaeal TatCs; blue, bacteria (except cyanobacteria); purple, mitochondria; and green, cyanobacteria and plastids; α, *Rickettsia* (closest relative to mitochondria); the inset shows the bootstrap-implemented tree. Most nodes are supported by high bootstrap values, indicating a generally robust and reliable tree topology. Low support values at several deep bacterial nodes indicate limited confidence in the resolution of these branches. However, this is irrelevant for the purpose of this tree.

Interestingly, the extreme acidophiles among the archaea evolved TatC variants that clearly escape the organismic phylogeny grouping (Fig. 5). All these archaea have optimum growth below pH 3, and some of them even below pH 1. To protect the cytoplasm from uncontrolled entry of protons at such growth conditions, the ΔΨ at the cytoplasmic membrane is very low or even inverted in this extremely acidophilic archaeal group, with the outside being negative and the inside positive (Baker-Austin and Dopson 2007, Schäfer et al. 1999) (Fig. 6A). This has an enormous impact on the Tat mechanism: In an inverted electric field at extremely low pH, the negative charge would inhibit a required movement of the TM5/TM6-domain. It is thus revealing to see that these extremely acidophilic archaea lost the negative charge in this region of the TM5/TM6 domain (Fig. 6C). In normal archaeal TatAC systems, the “D211”-equivalent charge is two positions away from the position found in bacteria, mitochondria and plastids, which may suggest an independent convergent evolution for the use of ΔΨ in archaea and bacteria, and hence a “post-LUCA” development (red dots in Fig. 6C). The absence of the conserved “D211” in extremely acidophilic archaea suggests that (i) these systems may function without using a ΔΨ, and that (ii) a selection against the negative charge in the “D211” position of the TM5/TM6 domain occurred due to a required movement of this domain.

**Figure 6:**
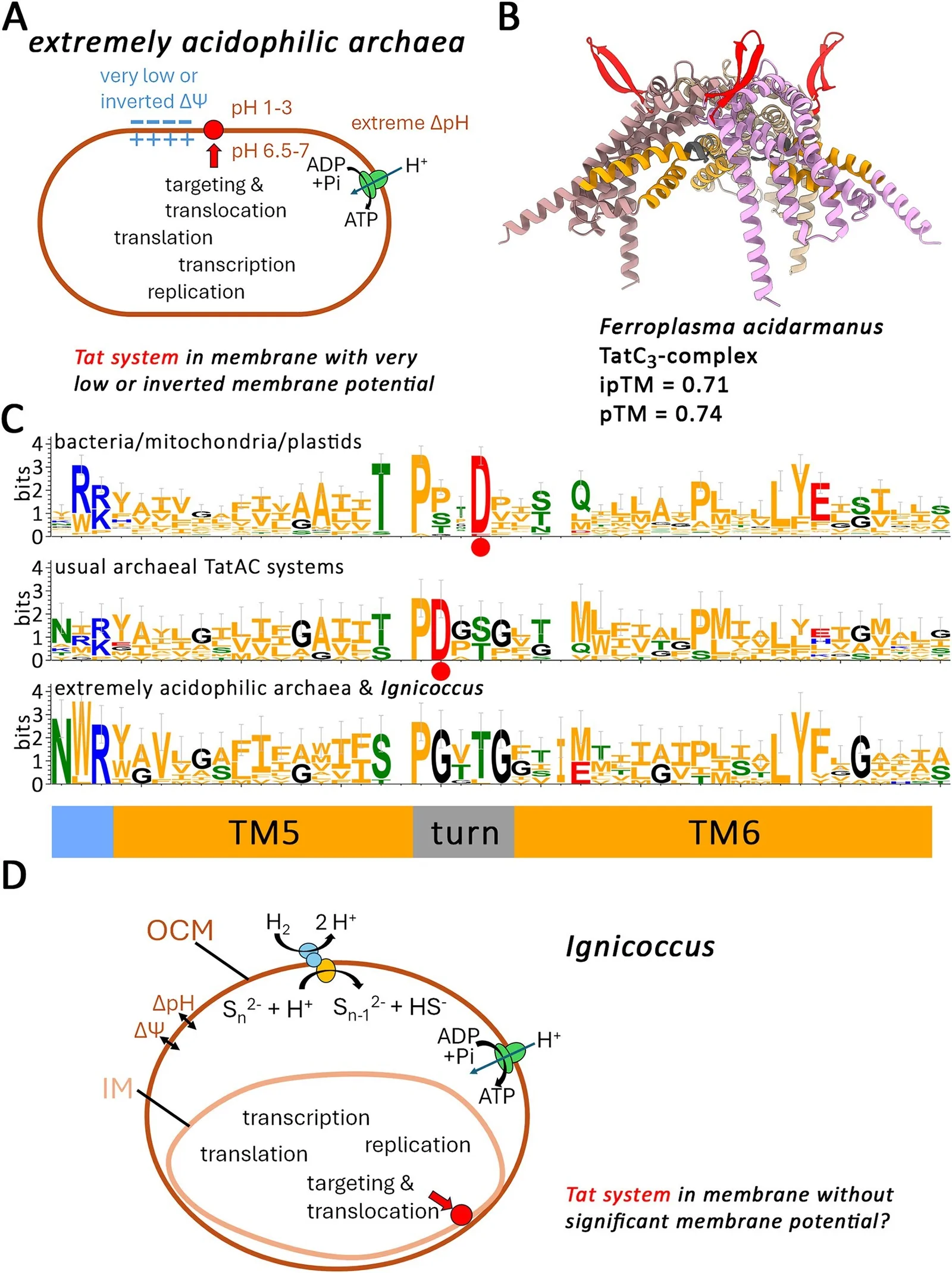
**Tat transport can function in membranes with no significant ΔΨ. (A)** Scheme of an extremely acidophilic archaeon, highlighting the enormous pH gradient and the inverted membrane potential at the cytoplasmic membrane. The A-type ATP synthase of these organisms is entirely triggered by the ΔpH. **(B)** AF3 model of a representative TatC_3_ complex from an extremely acidophilic archaeon, highlighting the hairpin at the end of TM1 (red), as well as the position of the TM5/TM6 (orange) with its connecting turn (grey). **(C)** WebLogo plots of TatC-TM5/TM6 domains of the TatC alignment used for the phylogenetic tree. The regions of TM5, the turn and TM6 are indicated at the bottom (colors as in **(B)**). Upper plot: TatCs from bacteria, mitochondria and plastids, middle plot: TatCs from normal archaeal systems, and lower plot: TatCs from extremely acidophilic archaea and *Ignicoccus hospitalis*. **(D)** Scheme of an *Ignicoccus* cell with its outer cellular membrane (OCM) and inner membrane (IM). Shown are also a key membrane-energizing ETC and the A-type ATP synthase in the OCM. No membrane energization is known for the IM, nor are ATP synthases in that membrane.

The electric field is thus unlikely to be strictly needed for the mechanism, although it is necessary for all model Tat systems that have been analyzed so far. But if TatC of extremely acidophilic archaea does not use a ΔΨ, how is unfolding at the mature region next to the signal peptide energized and what could trigger the suggested exposure of the TM5/TM6 domain at the surface to permit the docking of TatA? If the Tat substrates are not stably folded in the region next to the signal peptide, the binding energy may suffice to promote signal peptide insertion, and there can be several ways to achieve the coupling of signal peptide insertion to the exposure of the TM5/TM6 domain. Signal peptide insertion per se might be able to push this domain to the surface in phase I. Also, supportive structural adaptations may have evolved. We noted that the AF3-predicted TatC structures in these acidophilic archaea have unusual beta-sheet hairpins at the end of TM1, which might play such a role (Fig. 6B). Another aspect is the energy requirement in phase II, the TatA-dependent membrane disruption. Here, the extreme pH gradient at the very stable and proton-tight cytoplasmic membrane of these acidophiles could facilitate the process, similar to the facilitation of the process by cell-penetrating peptides (McNeilage et al. 2023).

As a side aspect, we would like to note that TatCs of extremely acidophilic bacteria, unlike the extremely acidophilic archaea, are predicted to use the ΔΨ, as evidenced by their conserved “D211”. This is possible because these bacteria have to locally generate small inside negative membrane potentials, even though on average they may have an inverted membrane potential when growing below pH 3 (Bakker 1990). They need this ability, as bacterial F-type ATP synthases require at least a small but normal oriented (negative inside) membrane potential, even when artificially large proton gradients are imposed (Ballmoos et al. 2009, Dimroth et al. 2000, Kaim and Dimroth 1999). It is thus not surprising that extremely acidophilic bacteria have the opportunity to use the membrane potential for Tat transport. The A-type ATP synthases of archaea do not strictly require a membrane potential and hence extremely acidophilic archaea likely do not establish local membrane regions with significant negative inside oriented membrane potentials (Gloger et al. 2015). This can be the reason why the Tat system in these archaea needs to function without the use of a membrane potential.

### The special case of *Ignicoccus*—another TatC that works without ΔΨ?

After finding that extremely acidophilic archaea lost the conserved aspartic acid (“D211”) in the TM5/TM6 domain, which points to an adaptation to an inverted membrane potential, we examined the conservation of this negatively charged position in the complete set of bacteria and archaea that we had used for the phylogenetic tree. To our surprise, we found only members of one other genus that showed the absence of this negatively charged position: *Ignicoccus* (Fig. 6C). The hyperthermophilic genus *Ignicoccus* differs from all other known prokaryotes by having an “outer cellular membrane” (OCM) that is proton-tight and energized by a redox-driven proton gradient for fueling ATP synthase at this membrane, whereas an “inner membrane” (IM) surrounds a compartment containing DNA and all information processing machinery (including replication, transcription, and translation) (Heimerl et al. 2017, Küper et al. 2010) (Fig. 6D). The exact function of the IM is yet unknown, but as the IM is not involved in energy transduction and ATP synthesis, it is likely that the IM is only weakly energized, which would explain the presence of a ΔΨ-independent Tat system in *Ignicoccus*. The characteristics of the translocated substrates and the IM of this genus may demand only little energization for Tat transport phases I and II. As outlined above, one simple explanation for ΔΨ-independence could be the presence of an unfolded region at the N-terminus of the mature domain in Tat substrates, making ΔΨ-energized unfolding that depends on “D211” unnecessary. However, this hypothesis needs to be experimentally addressed in future.

In view of the unusual abilities of Tat systems in extremely acidophilic archaea and *Ignicoccus*, the in other organisms usually observed ΔΨ-energization of phase I can be an evolutionary adaptation of Tat systems to the advantageous use of the existing significant ΔΨ in those membranes. For the pmf- or smf- dependent energization of phase II, which is much less understood, small chemical gradients (ΔpH, ΔNa^+^) may suffice, depending on the membrane rigidity (Bageshwar and Musser 2007).

### A protective function of membranes and Tat systems in early evolution?

It is simple to say that membranes “evolved” and that initial coincidental abiotic leaky membranes were over time substituted by synthesized membrane lipids, but the selective pressure needs to be rationalized, the advantage that led to the evolution of membranes and Tat transport. This initial selective pressure could not have been the use of membranes for the generation of proton or sodium gradients to generate ATP, because this bioenergetic use requires the synthesis, targeting, and functional assembly of a highly complex nanomachine called ATP synthase, which evolved by a combination of a hexameric P-loop ATPase, a membrane-integral ion channel, and other interaction partners in the membrane (Mahendrarajah et al. 2023, Mulkidjanian et al. 2007). Earlier simpler systems could have been based on ion-gradient driven pyrophosphate syntheses (Baykov et al. 2022). In any case, ancient SRP/SecY/YidC-assisted pathways were needed for the evolution of ion gradient-utilizing membrane-integral synthases of high-energy products. It is believed that, after an initial use of natural gradients, membrane-associated electron transport chains (ETCs) evolved, which led to the self-determined generation of ion gradients that was a prerequisite for the liberation of the first cell from its presumed mineral cavity deep in an alkaline vent of the sea (Fig. 4B) (Goldman et al. 2023). Tat transport surely played a crucial role for these first ETCs, such as in the electron flow from hydrogen to polysulfides or N-oxides, as enzymes like nickel-containing hydrogenases needed to be Tat-dependently placed on the outside of the membranes for these ETCs (Gross et al. 1999, Rodrigue et al. 1999). In fact, hydrogenases were the first Tat substrates, whose translocation was biochemically shown, long before the pathway was discovered, with two interacting subunits being transported by means of only one signal peptide (van Dongen et al. 1988).

However, initial selective advantages of membrane evolution in pre-LUCA times must have been immediate effects on the cellular processes. We suggest that—besides a function of membrane compartments in the capture of synthesized biomolecules—membranes protected against harmful substances, especially the thiol- and Fe-S-cluster-reactive arsenite, which could inactivate key enzymes and cofactors that were needed for ancient biosynthetic pathways (Kirschning 2026, van Lis et al. 2013).

The important detoxification-aspect needs here some more detailed discussion, as it directly relates to the evolution of the Tat system. Arsenite is abundant in submarine geothermal habitats such as black smokers or alkaline vents, which are currently discussed as most likely habitats of pre-LUCA evolution (Martin 2011). Due to its thiol- and Fe-S-cluster reactivity, arsenite detoxification by oxidation likely played an important role in early evolution (Lebrun et al. 2003). Aio-type arsenite oxidases were the only arsenite oxidases that were present in LUCA (Szyttenholm et al. 2020). Today’s Aio-type arsenite oxidases detoxify arsenite outside the cytoplasm. Before membranes existed, Aio-type arsenite oxidases may have already been present to protect thiol-involving metabolism. They are minimally composed of two subunits, the catalytic subunit with a molybdopterin cofactor and an [3Fe-4S]^1+/2+^ cluster, and the Rieske subunit with its [2Fe-2S]^1+/2+^ cluster (Watson et al. 2017). The Rieske subunit can be regarded as ancestor of Rieske proteins of cytochrome *bc_1_*/*b_6_f* type complexes (Lebrun et al. 2003), while the catalytic subunit belongs to the dimethylsulfoxide (DMSO) reductase family and was likely an ancestor of today’s molybdopterin-containing terminal reductases (Szyttenholm et al. 2020). Aio-type arsenite oxidases have a Tat signal peptide at the N-terminus of their Rieske subunit, and arsenite oxidases are thus translocated as a heterodimer by use of a signal peptide of the small subunit (van Lis et al. 2012), which parallels the arrangement found in heterodimeric hydrogenases but is unusual for molybdopterin-containing Tat substrates (Rodrigue et al. 1999, van Dongen et al. 1988). The signal peptide of the Rieske protein likely anchored the first Aio-type arsenite oxidases to the outer face of the membrane, a topology that is still found in many Aio-type arsenite oxidases today (Duquesne et al. 2008, Lebrun et al. 2003, Prasad et al. 2009). Interestingly, membrane-anchoring by an uncleaved Tat signal peptide is evolutionarily conserved in Rieske proteins of cytochrome *bc_1_*/*b_6_f* type complexes (Dibrova et al. 2013).

Due to unfavorable redox potentials, arsenite oxidation cannot be coupled to CO_2_ reduction or polysulfide reduction (Szyttenholm et al. 2020). Arsenite oxidases were therefore probably reoxidized by electron flow to early N-oxide reductases. N-oxides, especially nitrate and nitrite, are considered to have been abundant in pre-LUCA times and the only high-potential electron acceptors available in archaean oceans before the appearance of significant free oxygen (Ducluzeau et al. 2009). Nitrate-dependent anaerobic arsenite oxidation still exists today (Wang et al. 2017). The evolutionary oldest N-oxide reductases are those of the Nar-type (Stolz and Basu 2002), which have cofactors highly similar to those of arsenite oxidases. Thus, an electron flow from arsenite at the outside to nitrate in the inside of the early cells may have been a first ETC at membranes, which served as a detoxification pathway. This pathway had an immediate positive effect on the cells, prior to the evolution of an ATP synthase that could use ΔpH or ΔNa^+^ for ATP synthesis, and prior to the evolution of known ancient ETCs that served the purpose of self-controlled ion gradient formation, such as electron flow from hydrogen to polysulfides, which were likely present in abundance in hydrothermal vents. Later in evolution, when more oxidized compounds appeared on earth, alternative terminal electron acceptors could be used and new respiratory pathways emerged. Especially more extracytoplasmic Tat-dependently translocated molybdopterin-containing oxidoreductases evolved, which permitted the use of a multitude of alternative terminal electron acceptors for diverse anaerobic respirations that are known today, such as arsenate, selenate, thiosulfate, tetrathionate, polysulfide, perchlorate, chlorate, dimethyl sulfoxide, or trimethylamine N-oxide respirations (Wells et al. 2020). Beside these molybdoproteins, a wealth of other Tat-dependently translocated cofactor-containing proteins—mostly but not always redox proteins—evolved that enabled not only many different respiratory and photosynthetic ETCs, but also other metabolic pathways or stress responses. This is why Tat transport is until today closely associated with the translocation of cofactor-containing proteins. However, it is not mechanistically restricted to cofactor-containing proteins and is used whenever a *general* translocation pathway for folded proteins is needed.

## Concluding remarks and future directions

As outlined in this review, the diversity of today’s Tat systems shows a remarkable adaptivity during evolution. Tat is much more than the initially believed ΔpH pathway (Henry et al. 1997). The electric potential at the membrane is likely used by Tat in most cases, but functional Tat transport without a significant membrane potential was made possible in extremely acidophilic archaea and some exceptional hyperthermophilic species with uncommon cellular compartments (Fig. 6). Considering the diverse Tat systems that evolved, it becomes conceivable that Tat transport is only expensive due to a requirement of a transient membrane disruption. Future studies will now need to focus on the postulated individual events during Tat transport and clarify the proposed molecular basis for the energetics as well as the structure and structural transitions of TatC-containing complexes and TatA assemblies. In principle, Tat systems can function at hardly energized membranes or even at membranes with an inverted membrane potential. Uncovering the bioenergetics of this phenomenon will be a fascinating future research goal. For the transient membrane disruption in phase II, a ΔpH or ΔNa^+^ is likely important, and small gradients may suffice, depending on the membrane characteristics. The understanding of these effects similarly demands advanced studies. Due to the unexpected adaptability of Tat systems to diverse energization conditions, TatC-based transport was possible very early in evolution, which explains why Tat systems date back to LUCA. The use of the membrane potential possibly evolved later in archaea and bacteria. In view of the energetic expenses, will Tat systems persist at energized membranes in future evolution? As long as there is no “cheaper” alternative that works with a broad range of folded proteins, Tat transport is the pathway without alternative. In that sense, Tat transport is always a compromise. It is like an old machine that is still used, because a better transport machine for multiple folded substrates has not yet been invented by nature.
